# Diverging patterns of introgression from *Schistosoma bovis* across *S*. *haematobium* African lineages

**DOI:** 10.1371/journal.ppat.1009313

**Published:** 2021-02-05

**Authors:** Olivier Rey, Eve Toulza, Cristian Chaparro, Jean-François Allienne, Julien Kincaid-Smith, Eglantine Mathieu-Begné, Fiona Allan, David Rollinson, Bonnie L. Webster, Jérôme Boissier

**Affiliations:** 1 Univ. Montpellier, CNRS, IFREMER, UPVD, IHPE, Perpignan, France; 2 Centre for Emerging, Endemic and Exotic Diseases (CEEED), Department of Pathobiology and Population Sciences (PPS), Royal Veterinary College, University of London, Hawkshead Campus, Herts, United Kingdom; 3 Wolfson Wellcome Biomedical Laboratories, Department of Life Sciences, Natural History Museum, London, United Kingdom; 4 London Centre for Neglected Tropical Disease Research, Imperial College London School of Public Health, London, United Kingdom; Western University of Health Sciences College of Osteopathic Medicine of the Pacific NW, UNITED STATES

## Abstract

Hybridization is a fascinating evolutionary phenomenon that raises the question of how species maintain their integrity. Inter-species hybridization occurs between certain *Schistosoma* species that can cause important public health and veterinary issues. In particular hybrids between *Schistosoma haematobium* and *S*. *bovis* associated with humans and animals respectively are frequently identified in Africa. Recent genomic evidence indicates that some *S*. *haematobium* populations show signatures of genomic introgression from *S*. *bovis*. Here, we conducted a genomic comparative study and investigated the genomic relationships between *S*. *haematobium*, *S*. *bovis* and their hybrids using 19 isolates originating from a wide geographical range over Africa, including samples initially classified as *S*. *haematobium* (n = 11), *S*. *bovis* (n = 6) and *S*. *haematobium* x *S*. *bovis* hybrids (n = 2). Based on a whole genomic sequencing approach, we developed 56,181 SNPs that allowed a clear differentiation of *S*. *bovis* isolates from a genomic cluster including all *S*. *haematobium* isolates and a natural *S*. *haematobium-bovis* hybrid. All the isolates from the *S*. *haematobium* cluster except the isolate from Madagascar harbored signatures of genomic introgression from *S*. *bovis*. Isolates from Corsica, Mali and Egypt harbored the *S*. *bovis*-like *Invadolysin* gene, an introgressed tract that has been previously detected in some introgressed *S*. *haematobium* populations from Niger. Together our results highlight the fact that introgression from *S*. *bovis* is widespread across *S*. *haematobium* and that the observed introgression is unidirectional.

## Author summary

Hybridization is a fascinating evolutionary phenomenon that raises the question of how species maintain their integrity. Inter-species hybridization occurs between certain *Schistosoma* species that can cause important public health and veterinary issues. In particular hybrids between *Schistosoma haematobium* and *S*. *bovis* associated with humans and animals respectively are frequently identified in Africa. Recent genomic evidence indicates that some *S*. *haematobium* populations show signatures of genomic introgression from *S*. *bovis*. Here we conducted a comparative genomic study to assess the genomic diversity within *S*. *haematobium* and *S*. *bovis* species and genetic differentation at the genome scale between these two sister species over the African continent. We also investigated traces of possible ancient introgression from one species to another. We found that *S*. *haematobium* display low genetic diversity compared to *S*. *bovis*. We also found that most *S*. *haematobium* samples harbor signature of past introgression with *S*. *bovis* at some genomic positions. Our results strongly suggest that introgression occurred long time ago and that such introgression is unidirectional from *S*. *bovis* within *S*. *haematobium*. Such introgresssion event(s) result in diverging patterns of genomic introgression across *S*. *haematobium* lineages.

## Introduction

Hybridization is ubiquitous within the tree of life that raises the question of how species maintain their integrity [1–3]. Although hybridization may often result in the production of non-viable or non-fertile offspring, increasing evidence suggests that certain interspecific crosses produce not only viable progeny, but those progeny may be more successful than their parental species, at least during the early generations (e.g. hybrid vigor) [4,5]). For longer evolutionary timescales, genomic introgression resulting from hybridization events may be adaptive when genes and/or alleles from one species are maintained through subsequent generations and backcrosses by natural selection, becoming part of the genomic background [4,6,7]. One classical example of adaptive introgression relates to our own modern human lineage that has acquired genes involved in important functions such as immunity, pigmentation or resistance to hypoxia, originating from at least two introgression events with related ancestral lineages including Neanderthals and Denisovans [6]. It is thus now generally acknowledged that hybridization constitutes an important fuel for evolution and adaptation [8].

Hybridization also occurs between parasites and might have dramatic evolutionary outcomes [9]. In particular, several studies suggest that interspecific crosses among parasite species can generate lineages that display increased virulence compared to parental species, possibly leading to atypical pathologies and/or a wider host spectrum [9–11] and hence new epidemiological dynamics [9,12]. Hybridization and introgression events in parasites are thus likely to pose serious challenges for prevention, control and therapies against parasitic diseases [9].

Schistosomes are gonochoric parasitic flatworms with two successive hosts, a vertebrate as the definitive host in which adult worms reproduce sexually, and a freshwater snail species in which parasites undergo clonal multiplication. *Schistosoma spp*. can cause chronic and acute intestinal or urogenital schistosomiasis posing serious public health and veterinary issues. In tropical and subtropical regions it is thought that 143 million people are infected with schistosomes, with an estimated 24 thousand people dying each year from related pathology (IHME; Global Burden Disease). The *Schistosoma* genus diverged approximately 22 million years (my) ago and is comprised of 23 known species that specifically infect a broad range of mammals including Carnivora, Cetartiodactyla, Perissodactyla, Rodentia and Primates, including humans, as definitive hosts [13,14]. Inter-species hybridization is known to occur between certain *Schistosoma* species, with experimental inter-species crosses demonstrating important variation in the resulting offsprings’s fitness dependent on the species involved [15]. Natural inter-species hybrids have also been recognized between closely related sister species (e.g. *S*. *haematobium* and *S*. *guineensis*) [16], but also between more distantly related species that diverged up to ~12.5 my ago (e.g. between *S*. *mansoni* and *S*. *haematobium*) [17], that reside in the same definitive host. However, the evolutionary distance of the species involved does dictate hybrid viability. Hybrids resulting from closely related species that have different mammalian host preferences (e.g. non-human animals versus humans) are generating much research attention due to potential zoonotic consequences. For instance, *S*. *bovis* infecting ungulates, especially livestock, and *S*. *haematobium* infecting humans diverged 3.3 my ago but can successfully interbreed as testified by empirical evidence from the field [18–20], and by experimental studies [18,21]. These hybrids between animal and human associated *Schistosoma* species have recently received considerable attention due to their ease of inter-breeding, geographically widespread sympatric transmission, high-prevalences and moreover due to the potential zoonotic implications that this hybridization system suggests. Additionally, host pathology cannot be disregarded and in this context, J. Kincaid-Smith [22] experimentally demonstrated hybrid vigor in the first generation hybrids between a *S*. *bovis* and *S*. *haematobium* laboratory cross. The hybrids were compatible with natural snail hosts of both parental species, (i.e. *Bulinus truncatus* and *Planorbarius metidjensis)* and were more virulent than the parental strains within the vertebrate definitive host (in this case the experimental rodent *Mesocricetus auratus*). Additionally, this hybrid lineage was found to have established itself outside its native tropical range; in Corsica (France), where natural populations of *B*. *truncatus* occur. The hybrids established in Corsica display a highly introgressed genome, 77% was of *S*. *haematobium* origin, and 23% of *S*. *bovis* origin [21]. In this context, it was also hypothesised that this introgression may have enabled the establishment of urogenital transmission outside of its typical endemic zones [19].

A recent genomic study showed that *S*. *haematobium* populations from Niger were introgressed with a small proportion of *S*. *bovis* genomic background (i.e. ~3–8%) [23]. The authors suggest that this likely results from ancient hybridization and that the resulting introgression might have been adaptive with strong directional selection of a specific adaptive gene (i.e. *Invadolysin*) related to host-pathogen interactions originating from *S*.*bovis* and becoming fixed in these introgressed populations. Interestingly, this introgressed adaptive gene was also identified in the reference genome of *S*. *haematobium*, originally sampled in Egypt and now reared as a laboratory strain for over half a century [23,24]. These recent findings challenge the genetic integrity of the species *S*. *haematobium* and call into question whether pure *S*. *haematobium* lineages actually persist in Africa. Further genomic analyses are thus required to best characterize *S*. *haematobium* populations and unravel the genomic relationships between *S*. *haematobium*, *S*. *bovis* and their hybrids across all endemic countries.

**Fig 1:**
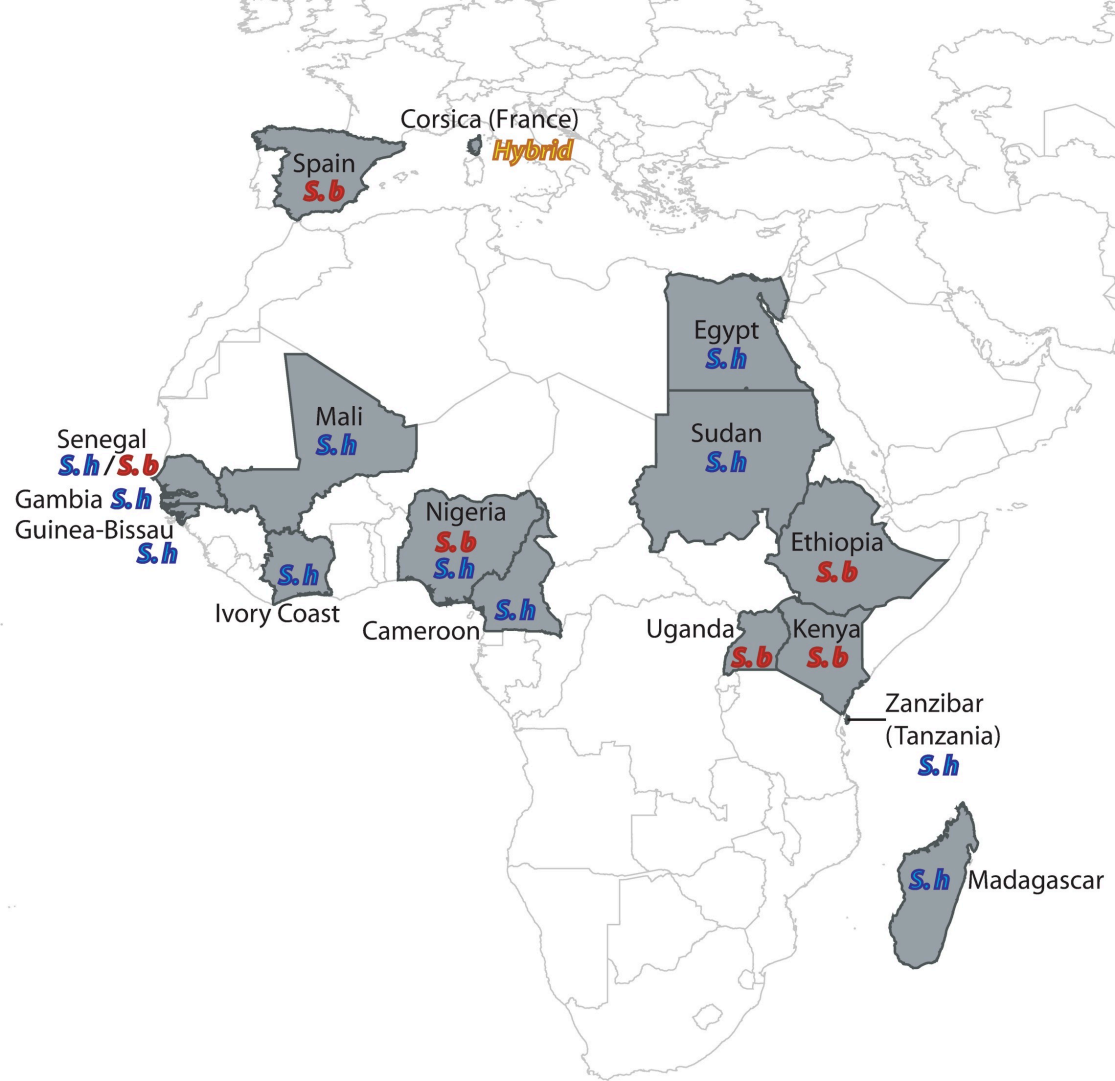
Geographical origins of the *S*. *haematobium* (S. h), *S*. *bovis* (S. b) and hybrid samples analyzed in this study.

In this context, we performed genome-wide analyses based on *Schistosoma* samples originating from a wide range of East and West African countries and initially characterized as *S*. *haematobium*, *S*. *bovis* or *S*. *haematobium—S*. *bovis* hybrids as well as an experimentally generated *S*. *haematobium*-*bovis* F1 hybrid (Fig 1; Table 1). Specifically, we developed a set of genome-wide SNPs from whole genome sequencing of 19 isolates to study the genomic relationship between the two parental species *S*. *haematobium* and *S*. *bovis* and their hybrids. Based on these SNPs we investigated possible traces of introgression events between these two sister species.

**Table 1:** Sample information. Samples from countries in bold were obtained from SCAN [25].

Species affiliation	Country	Collection year	Live stage at collection	Host retrieved from
***S*. *bovis (S*.*b)***	**Kenya**	1985	*Cercariae*	*B*. *tropicus*
	**Nigeria**	1988	*Cercariae*	*B*. *truncatus*
	**Senegal**	2000	*Miracidia*	Cow
	**Uganda**	2006	*Cercariae*	Undefined snail
	Ethiopia	2018	Adult	Cow
	Spain	Lab.strain*	Adult	Hamster
***S*. *haematobium (S*.*h)***	Egypt	Lab.strain*	Adult	Hamster
	**Cameroon**	2001	*Miracidia*	Human
	**Gambia**	1987	*Cercariae*	Undefined snail
	**Guinea-Bissau**	1990	*Cercariae*	*B*. *senegalensis*
	**Madagascar**	1987	*Miracidia*	Human
	**Nigeria**	1986	*Cercariae*	*B*. *truncatus*
	**Sudan**	1984	*Miracidia*	Human
	**Mali**	1994	*Cercariae*	Undefined snail
	**Tanzania (Zanzibar)**	1994	*Miracidia*	Human
	IvoryCoast	2018	*Miracidia*	Human
	Senegal	2019	*Miracidia*	Human
***S*.*b—S*.*h hybrid***	F1 Hybrid	Lab.strain**	Adult	Hamster
	Corsica	2013	*Miracidia*	Human

* The laboratory strains from Spain and Egypt were maintained in the culturing facilities at the University of Perpignan, previously provided by Ana Oleaga [54] and the Biomedical Research Institute, Rockville, Maryland [55], respectively.

** The F1 hybrid was generated in 2015 by crossing a female *S. bovis* from Spain and male *S. haematobium* from Cameroon (Barombi-Kotto) [21].

## Material and methods

### Sampling collection

The sampling strategy consisted of sampling individual adult male worms which had been isolated from a range of African countries, to capture as much genomic diversity as possible in both what we consider to be *S*. *haematobium* and *S*. *bovis*. To accomplish this aim, we combined samples recently collected in the field (N = 4), historical samples from the SCAN project (N = 12) [25] and samples from laboratory strains (N = 3) including one *S*. *haematobium* sample from Egypt and one *S*. *bovis* sample from Spain, both lineages having been passaged in the laboratory for more than 30 years. The Spanish *S*. *bovis* isolate was used for assembling the reference genome of *S*. *bovis*. The remaining laboratory strain consisted of a *S*. *haematobium-bovis* F1 hybrid resulting from the cross between a female *S*. *bovis* (Spain) and a male *S*. *haematobium* (Cameroon) generated at the University of Perpignan animal facility [21]. Our final sample set consisted of 19 adult males that were either collected as adults or originally as free-living stages (Table 1). If isolated from larval stages, adult worms were obtained from laboratory life-cycles using hamsters (*Mesocricetus auratus*) for *S*. *haematobium* or mice (*Mus musculus*) for *S*. *bovis* as the definitive hosts.

### Genomic DNA extraction

Total gDNA was extracted from each individual using the Qiamp DNA Micro Kit (Qiagen), following the protocol for “isolation of genomic DNA from tissues” and ultimately eluted in 25 μl of distilled water. The concentration and quality of each individual gDNA extract was assessed using a Qubit 3.0 fluorometer (Invitrogen).

### Library preparation and genome-wide sequencing process

The supposed nature of each isolate (i.e. *S*. *haematobium*; *S*. *bovis* or hybrid) was first validated based on a classical genotyping at the mitochondrial *COI* and at the *ITS2* nuclear gene [18,26,27]. Individual genomic libraries were prepared using the Nextera XT DNA Library Prep Kit (Illumina). One nanogram of genomic DNA was used as the template for each individual sample. Libraries were cleaned using 90 μL of AMPure XP beads (Beckman Coulter) to enrich libraries with fragments ranging from 300 to 500 bp. The quality of each library was then assessed using Agilent Technology 2011 Bioanalyser with a High Sensitivity DNA chip as recommended in the protocol. Individual libraries were sent to the Bio-Environment platform (University of Perpignan) where they were normalized and pooled before being paired-end sequenced within two independent runs (2x150 bp) on a NextSeq 550 (Illumina).

### Development of genome-wide SNPs

Demultiplexed FASTQ R1 and R2 files were uploaded to the Galaxy web platform [28]. R1 and R2 read datasets for each sample were first compiled into a single list of dataset pairs. Raw data quality within each list was assessed using the FastQC tool [29]. Reads were then trimmed to remove low quality sequences and the sequencing and index adapters. Once cleaned, reads were aligned to the *S*. *haematobium* (Egyptian strain) reference genome ShV2_May19_020001-666.fa (of 370 Mb and which contains 666 scaffolds; available on GenBank BioProject PRJNA78265) using bowtie2 [30] using default parameters settings. Aligned reads from each sample were merged together in a unique.BAM file and alignment statistics were retrieved using the Samtools flagstat tool [31]. The total numbers of reads before and after preprocessing together with individual alignment rate are provided in S1 Table.

SNP calling was performed using GATK 4.0. [32]. Within GATK the HaplotypeCaller tool was first used on individual.BAM files for each sample. The resulting individual “.gvcf” files were next combined into a unique“.gvcf” file using the CombineGVCFs tool. SNPs were then filtered using the VariantFiltration tool and setting the following filtering parameters: FS > 45.0; QD < 2.0; MQ < 40.0; MQRankSum < -12.5 and ReadPosRankSum <-8.0. Finally, the genotyping file was obtained by filtering genotypes using vcftools version 0.1.16 [33] based on the former filtering parameters (—remove-filtered-all), removing indels (—remove-indels), prohibiting any missing data (—max-missing 1), keeping SNPs with minimal depth of 8 (—minDP 8) and minor alleles present in at least 3 copies (—mac 3). Finally, from the initial 57,615 SNPs obtained from GATK, only biallelic sites were conserved and all SNPs belonging to the mitochondrial genome (i.e. “020257” in the genome of reference) were removed to specifically focus on the nuclear genome. These filtering steps resulted in a set of 56,181 SNPs that were used for subsequent analyses.

### Genomic analyses

#### Clustering analysis

To analyse the global structure of genetic variation, a principal component analysis (PCA) was performed based on the 56,181 filtered SNPs using the dudi.pca command from the “ade4” package [34] implemented in R version 4.0.2 [35]. Isolates were plotted according to their coordinates on the first two PCA axes using the “factoextra” package in R [36]. To detect possible finer genetic structure among isolates, three additional PCA plots were performed to represent the spatial distribution of each isolates within three other dimensional spaces including the two following PCA axes (i.e. Axes 2 and 3; Axes 2 and 4 and Axes 3 and 4).

#### Within and between species genomic diversity

Based on the 56,181 initial filtered SNPs, individual observed heterozygosity (Hobs) was first calculated for each isolate. Difference in mean Hobs between species was assessed using a Mann-Whitney test in R. According to the results from the clustering analysis (see results), the six nominal *S*.*bovis* isolates were assigned to a *S*. *bovis* cluster, and the 11 nominal *S*. *haematobium* isolates together with the hybrid isolate from Corsica were assigned to a *S*. *haematobium* cluster. The same assignment of isolates among the two defined clusters was set and the F1 hybrid generated in the laboratory was excluded from all subsequent analyses. Second, the average pairwise nucleotide difference among isolates (Π) was computed within each cluster and pairwise nucleotide differences computed at each site (N = 56,181) were compared between the *S*. *bovis* and *S*. *haematobium* clusters, using a non-parametric paired Wilcoxon test in R. Third, Tajima’s Ds were estimated for each species. To statistically compare Tajima’s D between species, a series of Tajimas’Ds was computed along non-overlapping 50-kb sliding windows overall genomic scaffolds within each species. Windows that contained less than eight SNPs were discarded and only the 50-kb sliding windows in which Tajima’s Ds could be estimated for *S*. *bovis* and *S*. *haematobium* (i.e. with at least one segregating site within each species) were kept (N = 4035). The series of estimated Tajima’s Ds obtained for each species were next compared using a paired Wilcoxon test. Finally, overall absolute genomic divergence (Dxy) between *S*. *bovis* and *S*. *haematobium* was estimated. All nucleotide diversity and divergence estimates were computed using the ‘PopGenome’ R Package [37].

#### Spatial genomic structure within the *S*. *bovis* and *S*. *haematobium* clusters

To investigate any potential spatial genetic structure among isolates within each species, we computed pairwise Nei’s genetic distances matrices [38], between each isolate within each species (i.e. *S*. *haematobium* = all 11 nominal *S*. *haematobium* isolates + the Corsican hybrid isolate; and *S*. *bovis* = the six nominal *S*. *bovis* isolates), based on the initial filtered 56,181 SNPs and using the dartR package [39]. Correlation between the resulting matrices of pairwise Nei’s distances and pairwise geographical distances computed between the geographical origin of each isolate were next tested within each species using two independent Mantel tests as implemented in the ‘ape’ package [40] in R. Significance of correlations were assessed by comparing the obtained Z-scores to 10,000 Z-values generated after bootstrapping. Because uncertainty existed concerning the precise sampling location for some isolates, we considered the centroid of each country of origin obtained from QGIS v. 2.8.12 (QGIS Development Team).

#### Investigating signatures of introgression between *S*. *bovis* and *S*. *haematobium*

Introgression events are expected to result in the acquisition of genomic tracts from one species to another that can persist -and thus be detectable- within the introgressed lineage over time. This is dependent on time since the introgression event, the genomic context (e.g. genomic locality) of the foreign DNA tracts in the introgressed genome, the recombination rate, the selective advantage of the introgressed DNA tracts [1,3]. Using a genomic sliding windows approach, we thus investigated the presence of genomic regions that display significantly decreased divergence between each isolate and all isolates from its sister species (i.e. one versus all). Thus, to detect possible genomic tracts that could have been introgressed from *S*. *bovis* in each of the *S*. *haematobium* isolates, we computed Dxy estimates between each *S*. *haematobium* isolate (including the hybrid Corsican lineage) and all the *S*. *bovis* isolates along 50-kbs windows every 10 kbs overall genomic scaffolds. The same genomic windows were used to compute Dxy estimates between each *S*. *bovis* isolate and all the *S*. *haematobium* isolates (including the hybrid Corsican lineage) to detect potential traces of introgression from *S*. *haematobium* in each of the *S*. *bovis* isolates. The Dxy computations along the 50-kbs sliding windows were performed using the R package ‘PopGenome’ [37]. All 50 kbs-windows that contained less than eight SNPs were discarded for subsequent analyses. It was considered that a sliding window displayed significantly decreased divergence when the Dxy value computed was intrinsically lower than the lowest values (0.1%) obtained overall the windows (with > eight SNPs) and overall the isolates (N = 239,076; i.e. 13,282 windows x 18 isolates). This 0.1% threshold corresponded to a Dxy value of 0.102.

Along each scaffold, unique genomic tracts were reconstructed from all overlapping sliding windows that displayed significantly decreased divergence according to the above 0.1% threshold including the windows with less than eigth SNPs. The absolute size, the total number of SNPs and individual Dxy values (i.e. Dxy computed between each isolate and all isolates from its sister species) were computed for each genomic tract. Finally, we investigated the presence of potential coding genes within the resulting genomic tracts based on the ShV2_May19_SwissProtAnno.gff3 annotation file [41] and using IGV 2.3.92 [42].

## Results

### Clustering analysis

**Fig 2:**
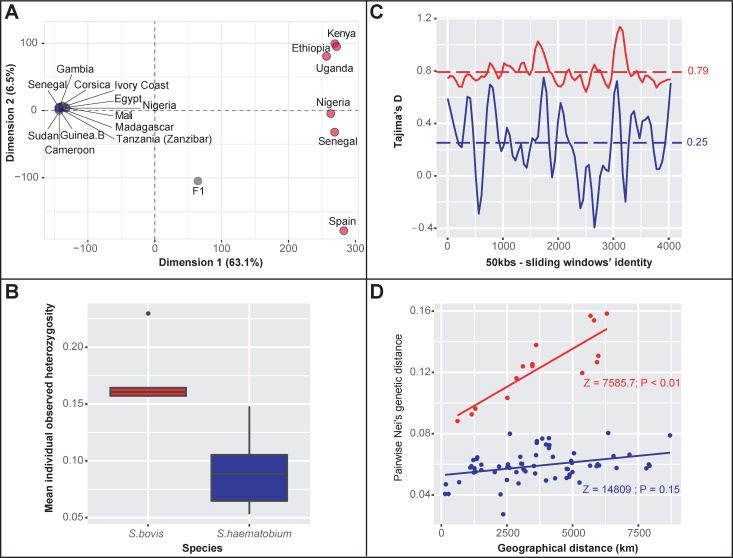
Genome-wide genetic diversity and genetic differentation within *S*. *haematobium* and *S*. *bovis*. **A.** Spatial representation of all isolates in a PCA dimension defined along the two first axis that explain 63.1% and 6.5% of the total genomic variance respectively. Isolates identified as *S*. *haematobium* are in blue, *S*. *bovis* isolates are in red, the Corsican hybrid is in yellow and the experimentally bred F1 hybrid is in grey. **B.** Mean individual heterozygosity within *S*. *bovis* (in red) and *S*. *haematobium* (including the Corsican lineage; in blue). **C.** Tajimas’D estimates along 4035 50 kbs-sliding windows that contained at least 8 SNPs over 159 scaffolds computed within *S*. *bovis* (in red) and *S*. *haematobium* (in blue). **D.** Plot of Nei’s distances computed between isolates and geographical distances between their countries of origin (using the countries’ centroid as proxy for each locality) within *S*. *bovis* (in red) and *S*. *haematobium* (in blue). The genomic structure among *S*.*bovis* lineages follows a classical significant isolation-by-distance pattern at the African continent scale.

Overall, 56,181 biallelic SNPs distributed over 239 of the 665 nuclear genomic scaffolds (i.e. ~ 36%) of the *S*. *haematobium* reference genome (Egyptian strain) were developed into a high-quality SNP dataset. Using this dataset, we could clearly distinguish between isolates categorized as either *S*. *haematobium* or *S*. *bovis* based on a principal component analysis (i.e. PCA; Fig 2A). In this regard, the overall absolute genomic divergence between *S*. *bovis* and *S*. *haematobium* reaches 0.24. As expected, the laboratory generated first generation *S*. *haematobium-bovis* hybrid (F1) fell mid way between the two parental strains used for its construction, i.e. *S*. *bovis* from Spain and *S*. *haematobium* from Cameroon in the PCA two-dimensional space (Fig 2A). No other sample displayed such an intermediate genomic pattern. More surprisingly, according to the first and second PCA axes (respectively representing 63.1% and 6.5% of the overall variance), all *S*. *haematobium* samples and the hybrid sample from Corsica, grouped into a unique genetic cluster (Fig 2A). The projection of all isolates in the other PCA two-dimensional spaces defined from the third and fourth PCA axes lead to the same pattern with a single condensed cluster with no genomic distinction of the hybrid Corsican isolate from the other *S*. *haematobium* isolates (S1 Fig).

### Within and between species genomic diversity

Our results indicate that the *S*. *haematobium* cluster as defined based on the PCA analysis (i.e. all 11 nominal *S*. *haematobium* and the Corsican hybrid isolates) harbored less genomic diversity than the *S*. *bovis* cluster. First, the average level of individual observed heterozygosity (Hobs) was significantly lower in the *S*. *haematobium* cluster (H_obs_ = 0.07) than in the *S*. *bovis* cluster (H_obs_ = 0.17; W = 0; P-value < 0.01; Fig 2B). Additionally, the mean pairwise nucleotide divergence per site is also lower in the *S*. *haematobium* compared to the *S*. *bovis* cluster (0.10 *versus* 0.18, V = 566942560; P-value < 0.01). Tajima’s D values computed along the genome among the *S*. *haematobium* isolates (including the Corsican lineage) were lower (although still positive) compared to those computed among the *S*. *bovis* isolates (0.29 *versus* 0.78; V = 5738688; P-value < 0.01; Fig 2C). Finally, pairwise genetic differentiation estimates between isolates within the two clusters were systematically lower in *S*. *haematobium* compared to *S*. *bovis* and while the genetic diversity among *S*. *bovis* isolates followed an isolation by distance pattern (Z = 7585.7; P-value< 0.01), no such pattern was found among *S*. *haematobium* isolates (Z = 14809; P-value = 0.15; Fig 2D).

### Signature of introgression between *S*. *bovis* and *S*. *haematobium*

**Fig 3:**
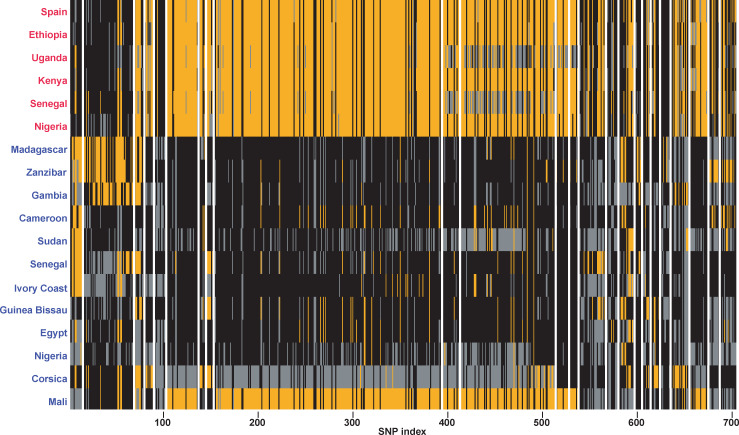
Representation of the allelic state of each isolate at each SNP (N = 705) along the *S*.*bovis*-like genomic tracts detected in at least one *S*. *haematobium* isolate N = 24). Each vertical bar represents a single SNP position. SNPs at which isolates are homozygous and harboring an allele identical or different to the genome of reference (Egyptian strain) [41] are in black and orange respectively. SNPs at which isolates are heterozygous are in grey. Independent genomic tracts along scaffolds are separated by a vertical white line. Individuals initially characaterised as *S. bovis* and *S. haematobium* are in red and blue respectively.

Overall, Dxy values between each isolate and all isolates from its sister species (i.e. one versus all) were computed over 34,852 50-kbs sliding windows along 168 genomic scaffolds. From these initial 34,852 sliding windows, 13,282 contained more than eight SNPs from which 239,076 individual D_XY_ values were retrieved for all the 18 isolates (i.e. all isolates except the F1 laboratory hybrid). These windows covered 53% of the genome (196.51 Mb) and 159 genomic scaffolds. A total of 158 windows distributed over 18 genomic scaffolds harbored significantly decreased genetic divergence between at least one isolate and all isolates combined from the sister species according to our 0.1% threshold. Of these 158 windows, 11 had significant decreases in divergence between at least one *S*. *bovis* isolate and all *S*. *haematobium* isolates and 147 had significant decreases in divergence between at least one *S*. *haematobium* isolate and all *S*. *bovis* isolates. Finally, from these sliding windows a total of 29 genomic tracts (i.e. successions of adjacent sliding windows) characterized by extremely low genomic divergence were reconstructed with absolute sizes ranging from 2,456 bp to 630,020 bp and containing from eight to 241 SNPs. Twenty four of these genomic tracts corresponded to genomic regions that display low Dxy values computed between at least one *S*. *haematobium* isolate and all *S*. *bovis* isolates (Table 2). These genomic tracts were 82,464 bp long on average (ranging from 9,620 bps to 630,020 bps) and contained on average of 23.4 SNPs (from eight to 241, Table 2; Fig 3). The remaining five genomic tracts corresponded to regions that display low Dxy values computed between at least one *S*. *bovis* and all the *S*. *haematobium* isolates combined (including the Corsican hybrid isolate; Table 3). These five genomic tracts were on average 25,019 bp long (ranging from 2,456 to 48,670 bp) and contained on average of 9.2 SNPs (from eight to 13; Table 3). Finally, over the 24 *S*. *bovis*-type genomic tracts found in at least one *S*. *haematobium* isolate, 36 genes were identified. In this regard, one genomic tract of length 212,351 bps from the scaffold 020018 contains, among other genes, the *Invadolysin* gene. Finally, only three genes were found within the five *S*. *haematobium*-type genomic tracts that were found in at least one of the *S*. *bovis* isolate (S2 Table).

**Table 2:** Characteristics of the 24 *S*. *bovis*-like genomic tracts detected in at least one *S*. *haematobium* isolate and Dxy values computed between each *S*. *haematobium* isolate and all the *S*. *bovis* isolates within each of these 24 *S*. *bovis*-like genomic tracts. The scaffold 020018 in bold and framed in the table contains the *S*. *bovis*-like *Invadolysin* gene (Fig 4). Red coloration levels reflect small (dark red) to high (light red) Dxy values. Extreme low Dxy values (i.e. <0.102) are in bold.

Scaffold identity	Number of SNPs	Start position	End position	Tract size (bp)	Cameroon	Corsica	Egypt	Gambia	Guinea Bissau	Ivory Coast	Madagascar	Mali	Nigeria	Senegal	Sudan	Zanzibar
20002	15	86414	113838	27424	0.583	**0.061**	0.356	0.356	0.139	0.65	0.65	0.433	0.5	0.65	0.65	0.583
**20018**	54	8423425	8635776	212351	0.145	**0.077**	**0.085**	0.549	0.173	0.343	0.59	**0.076**	0.196	0.373	0.117	0.608
20018	11	8850176	8899446	49270	0.545	**0.061**	0.303	**0.061**	0.424	0.485	0.424	0.242	0.303	**0.061**	0.485	0.424
20040	10	1452306	1486703	34397	0.65	**0.083**	0.7	0.417	0.7	0.392	0.7	0.75	0.442	0.7	0.7	0.75
20062	13	5240139	5266732	26593	0.308	0.346	0.346	0.308	**0.077**	0.385	0.346	0.346	0.269	**0.077**	0.308	**0.077**
20072	34	1770	136940	135170	0.875	0.488	0.86	0.904	0.904	0.89	0.86	**0.071**	0.831	0.875	0.743	0.875
20120	8	315078	342710	27632	0.667	**0.042**	0.604	0.542	0.604	0.667	0.604	0.667	0.542	0.604	0.667	0.604
20120	8	1486323	1522893	36570	0.76	**0.094**	0.76	0.427	0.146	0.427	0.76	0.76	0.76	**0.094**	0.76	0.76
20135	241	9647	639667	630020	0.865	0.462	0.872	0.872	0.863	0.847	0.861	**0.018**	0.805	0.874	0.756	0.859
20135	19	651522	696944	45422	0.798	0.474	0.798	0.825	0.851	0.772	0.772	**0.096**	0.496	0.851	0.496	0.746
20135	101	813373	1147895	334522	0.77	0.382	0.771	0.743	0.757	0.755	0.724	**0.082**	0.606	0.767	0.521	0.768
20135	14	1223289	1270364	47075	0.869	0.798	0.798	0.798	0.798	0.798	0.869	**0.06**	0.833	0.798	0.798	0.94
20135	10	1314098	1323718	9620	0.817	0.817	0.817	0.817	0.817	0.817	0.817	**0.083**	0.817	0.817	0.817	0.817
20168	29	135	27437	27302	0.483	0.483	0.328	0.31	0.216	0.399	0.483	0.31	0.443	**0.086**	0.417	0.448
20168	13	1900169	1917247	17078	0.141	0.436	**0.09**	0.474	0.436	0.122	0.513	0.436	0.436	0.321	0.513	0.436
20173	17	1412975	1494068	81093	0.721	0.25	0.245	0.466	0.779	0.216	0.779	0.407	0.75	0.309	**0.093**	0.779
20173	8	6405980	6428204	22224	**0.073**	**0.073**	**0.073**	0.51	**0.073**	0.188	0.552	**0.073**	0.135	0.51	**0.073**	0.615
20174	9	1040987	1082699	41712	0.463	**0.074**	0.269	0.306	0.148	0.63	0.352	0.407	0.574	0.574	0.407	0.352
20174	10	2206173	2244270	38097	0.25	0.4	**0.1**	0.5	0.167	0.4	0.4	0.25	0.233	0.4	0.25	0.4
20175	11	419051	443512	24461	0.273	0.136	0.136	**0.045**	0.364	**0.091**	0.318	**0.045**	**0**	0.318	0.136	0.455
20241	20	361697	372646	10949	0.517	**0.1**	0.492	0.317	0.517	0.542	0.492	0.367	0.517	0.492	0.542	0.517
20241	20	1310224	1358966	48742	0.767	0.429	0.742	0.742	0.742	0.767	0.767	**0.092**	0.729	0.742	0.704	0.742
20243	12	821454	831590	10136	**0.063**	0.354	0.479	0.521	0.563	0.354	0.563	0.563	0.528	0.354	0.563	0.771
20243	18	890721	931992	41271	**0.097**	0.394	0.537	0.481	0.537	0.366	0.537	0.537	0.472	0.394	0.514	0.681

**Table 3:** Characteristics of the five *S*. *haematobium*-like genomic tracts detected in at least one *S*. *bovis* isolate and Dxy values computed between each *S*. *bovis* isolate and all the *S*. *haematobium* isolates within each of these five *S*. *haematobium*-like genomic tracts. Red coloration levels reflect small (in dark red) to high (in light red) Dxy values. Extreme low Dxy values (i.e. <0.102) are in bold.

Scaffold identity	Number of SNPs	Start position	End position	Tract size (bp)	Ethiopia	Kenya	Nigeria	Senegal	Spain	Uganda
20100	9	10330900	10379570	48670	0.313	**0.096**	0.591	**0.045**	0.48	0.146
20112	8	345456	347912	2456	0.568	**0.068**	0.506	0.443	0.631	0.256
20168	8	605485	645215	39730	0.193	0.506	0.131	0.688	**0.006**	0.756
20173	8	6220527	6240433	19906	0.341	0.301	0.403	**0.091**	0.182	0.301
20244	13	64302	78634	14332	0.105	0.259	**0.066**	0.259	0.643	0.182

## Discussion

### All *S*. *haematobium* isolates and the natural Corsican hybrid form a unique genomic cluster

Based on our clustering approach, we found that the hybrid isolate from Corsica, together with all nominal *S*. *haematobium* isolates, group into a unique cluster harboring low genomic diversity compared to the *S*. *bovis* cluster (Fig 2). This result was somewhat unexpected given that the Corsican isolate is classified as a *S*. *haematobium-bovis* hybrid based on discordant mt / nuclear genetic markers (*S*.*bovis*-type *CO1* mitochondrial haplotype and *S*. *haematobium*-type *ITS* nuclear haplotype). Moreover, we have previously shown the Corsican isolate’s entire mitochondria was of *S*. *bovis* origin, with an estimated 23% of its nuclear genome of *S*. *bovis* origin, whereas ~77% of *S*. *haematobium* origin, proving that this was not a first generation hybrid but that there had been introgression from *S*. *bovis* into *S*. *haematobium* [21]. Hence, we might have expected that this sample would have an intermediate location between the *S*. *haematobium* and the *S*. *bovis* clusters in the PCA space although closer to the *S*. *haematobium* cluster. Two non-exclusive main hypotheses might explain these results.

First, on a methodological perspective, our approach was based on the development of SNPs from supposedly orthologous regions between these two sister species. In particular, the initial sequencing reads obtained from each isolate were aligned to the *S*. *haematobium* reference genome (Egyptian strain) [41] and only those that aligned properly were considered for subsequent analyses. Kincaid-Smith et al. [21] assessed the introgression level of the Corsican strain using an independent analytical approach based on the alignment of sequencing reads on a concatenated genome combining the genomes of both parental species (i.e. *S*. *haematobium* and *S*. *bovis*). Sequences were next aligned according to the best mapping location over the two genomes and the proportion of sequences that aligned to the *S*. *bovis* and to the *S*. *haematobium* regions of the concatenated genome were then used to assess its introgression level. Using our approach, we might have missed some *S*. *bovis* introgressed genomic (private) regions in the Corsican isolate that did not align properly on the *S*. *haematobium* reference genome. Such private regions are expected to be rare due to the high genomic similarity between these two species [24]. Nevertheless, and if so, we might expect that using a *S*. *bovis* instead of a *S*. *haematobium* reference genome for the initial alignment of sequencing reads and for the development of SNPs would allow detection of such private *S*. *bovis* genomic regions and hence tease apart the Corsican isolate from the *S*. *haematobium* group. However, an additional PCA analysis conducted on SNPs developed from the alignment of the sequencing reads against the *S*. *bovis* reference genome, strictly led to the same conclusions (S2 Fig).

The second hypothesis is that all isolates originally described as *S*. *haematobium* are in fact introgressed, to some extent, with *S*. *bovis* similarly as with the hybrid isolate from Corsica. In this regard, two recent studies strongly suggest that at least two lineages initially identified as *S*. *haematobium* (based on morphological, ecological and genetic diagnostics), including the laboratory strain from which the reference genome was assembled (Egyptian strain) [41], were in fact introgressed with *S*. *bovis* although moderately (i.e. 3–8%) [23,24]. Based on these previous studies and because of the results obtained in the present study, we thus investigated the presence of genomic tracts from *S*. *bovis* that could have been acquired in the genomes of the *S*. *haematobium* isolates. We also investigated the presence of genomic tracts from *S*. *haematobium* within all the *S*.*bovis* isolates that could result from past introgression events between these two sister species.

### Evidence for past introgression events between *S*. *bovis* and *S*. *haematobium*

In total, 24 genomic tracts with extremely low levels of divergence between at least one of the *S*. *haematobium* isolate and all of the *S*. *bovis* isolates were identified (Table 2; Fig 3). These genomic tracts could result from their transfer from *S*. *bovis* to at least some *S*. *haematobium* lineages during past introgression events. Importantly however, a decrease in divergence between the two sister species is also expected locally at some genomic positions due to shared ancestral polymorphism even in the absence of introgression [2,43]. Admittedly, our current analyses do not account for such ancestral polymorphism. However, several results discredit this hypothesis in favor of a past introgression scenario.

**Fig 4:**
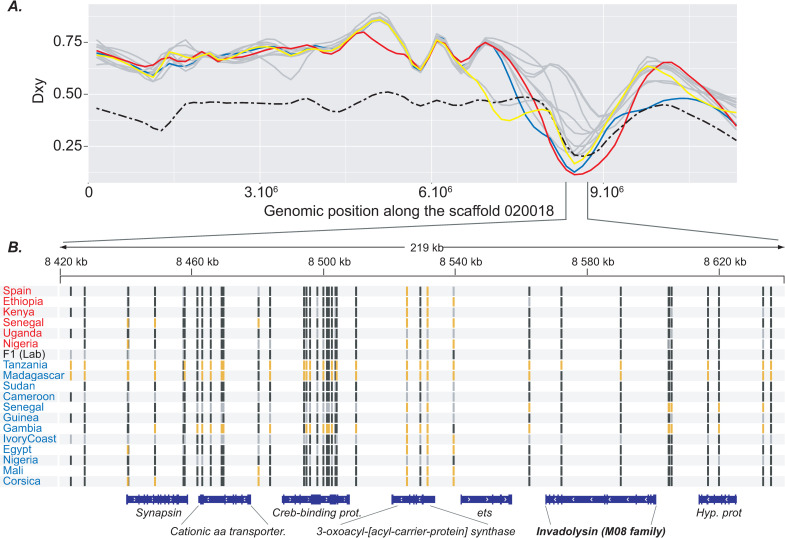
Focus on a *S*. *bovis*-like genomic tract detected in some *S*. *haematobium* isolates along the genomic scaffold 020018. **A.** Dxy values computed between each of the 12*S*. *haematobium* isolates and the experimental F1 hybrid and all of the *S*. *bovis* isolates along the genomic scaffold 020018. Extremely low values of Dxy were found between three *S*. *haematobium* isolates (i.e. Corsica, Mali and Egypt) and *S*. *bovis* according to the Dxy = 0.102 fixed threshold (see material and methods). Dxy values corresponding to the isolates from Corsica, Mali and Egypt are in blue, red and yellow respectively. Dxy values computed between the experimental F1 hybrid and all *S*.*bovis* isolates are represented by a dashed black line. Dxy values computed between all other isolates and *S*. *bovis* isolates are in grey. **B.** Representation of the allelic state of each isolate at each SNP along the 212.351 bp *S*.*bovis*-like genomic tract detected in the isolates from Corsica, Mali and Egypt that contain, among other genes, the *Invadolysin* gene previously identified in a *S*. *haematobium* population from Niger and in a *S*. *haematobium* reference strain (Egyptian strain) [41]. SNPs at which isolates are homozygous and harboring an allele identical or different to the genome of reference (Egyptian strain) [41] are in black and orange respectively. SNPs at which isolates are heterozygous are in grey.

First, the identified genomic tracts characterised by extremely low Dxy values were sometimes of considerable size (up to 630.020 bp) which is not expected under the retention of ancestral polymorphism scenario [44]. Second, contrary to what we found among *S*. *haematobium* isolates, very few genomic regions with significantly decreased divergence between each of the *S*. *bovis* isolates and all of the *S*. *haematobium* isolates were detected (N = 5). Under an ancestral polymorphism scenario we might have expected detecting reciprocal patterns of genomic similarities between each *S*. *bovis* isolate and *S*. *haematobium*. In this regard, our results thus not only support an introgression scenario between these two sister species but also that introgression was most likely unidirectional and has led to the transfer of some admixted tracts from *S*. *bovis* into at least some of the *S*. *haematobium* isolates. However, this latter conclusion needs to be taken with caution since only six *S*. *bovis* isolates were included in this study and we cannot rule out the existence of some *S*. *bovis* lineages introgressed with *S*. *haematobium* across Africa. Third, among the 24 *S*. *bovis*-like genomic tracts found over the *S*. *haematobium* isolates, one contains the *Invadolysin* gene (Table 2; Fig 4). This gene was previously identified to be of *S*. *bovis*-type in some introgressed individuals of a *S*. *haematobium* population in Niger and also in the isolate which is used as the *S*. *haematobium* reference [23]. In the present study, this same *S*. *bovis-*type sequence of the *Invadolysin* gene was found in the *S*. *haematobium* isolates from Egypt, Corsica and Mali. Moreover, *S*. *haematobium* isolates from Sudan, Cameroon and Guinea-Bissau also displayed very low Dxy values at this genomic tract although slightly higher than the arbitrarily fixed threshold (0.117, 0.145 and 0.173 respectively). Together, these results suggest that all of these isolates likely originate from the same ancient introgression event at the origin of the introgressed lineage from Niger identified previously that occurred ~108–613 generations ago according to Platt et al. [23].

Interestingly, the number of admixed genomic tracts greatly varies among the *S*. *haematobium* isolates. Isolates from Corsica and Mali respectively displayed 9 and 10 admixture tracts while all other isolates displayed up to 4 and the isolate from Madagascar none (Table 2). This result is particularly interesting since Madagascar is the only country sampled in this study in which *S*. *haematobium* lives in allopatry (i.e. where *S*. *bovis* was never detected). Accordingly, we argue that the isolate from Madagascar analyzed in the present study is the unique representative of what could be considered a pure *S*. *haematobium*. Zanzibar is another geographic region where *S*. *bovis* was considered absent until its first detection in 2016 [45]. Hence, we might have expected not to detect traces of potential introgression from *S*. *bovis* within this isolate. However, we did find a potential *S*. *bovis* admixture tract in the isolate from Zanzibar, which was also found in the isolates from Senegal and Guinea-Bissau. This result thus suggests that the *S*. *haematobium* from Zanzibar isolate analyzed in this study results from an old introgression event between *S*. *haematobium* and *S*. *bovis* and that the evolutionary history of this isolate is thus independent to the one from Madagascar. Importantly however, this unique potential admixture tract identified in the isolate from Zanzibar is relatively small (i.e. 26.593 bp) and contain few SNPs (N = 13; Table 2). Therefore, we cannot formerly rule out the possibility that this *S*. *bovis*-like genomic tract truly reflects a shared ancestral polymorphism rather than an introgression event between the two species.

Whether all *S*. *haematobium* isolates that have been identified as introgressed (i.e. all but the isolate from Madagascar) originate from a single common past introgression event between *S*. *bovis* and *S*. *haematobium* remains an unresolved question. On one hand, the fact that three isolates (six if considering Dxy values higher than the 0.102 threshold) among the 12 *S*. *haematobium* isolates studied displayed the same genomic tract that includes the *S*. *bovis*-like *Invadolysin* gene strongly supports a common introgression event at the origin of these isolates and the previously identified introgressed within *S*. *haematobium* from Niger [23]. On the other hand, most of the *S*. *bovis*-like genomic tracts identified within the isolates are generally specific to a single isolate only (Table 2). These later results suggest either a common introgression event that occurred a long time ago, which is in line with the single introgression event identified by Platt et al. [23] that has been dated to ~108–613 generations ago. Under this scenario, different genomic tracts including the one that contains the *Invadolysin* gene could have been fixed in some populations while lost in other populations through genetic drift. Alternatively, the observed heterogeneous pattern of admixed tracts from *S*. *bovis* within the *S*. *haematobium* isolates could reflect several independent past introgression events. Because the identified admixture tract, although generally longer than that expected under an ancestral polymorphism scenario, are relatively small in most *S*. *haematobium* isolates, the potential independent past introgression events are also likely to be ancient. Indeed, it is expected that successive recombination events over generations since the initial introgression event can lead to a drastic size reduction of admixture tracts in the resulting introgressed lineage [46].

On a final note, it is likely that the degree of genomic introgression from *S*. *bovis* to the *S*. *haematobium* isolates observed in this study is underestimated. In fact, only 53% of the overall genome was screened for potential genomic regions with a decrease of genomic divergence between these sister species in particular because we applied stringent filters applied for developing SNPs (i.e. no missing data) and we mainly focused on genomic regions with initial genomic variation (i.e. were SNPs were present in at least one isolate). This approach is likely to provide a conservative vision of introgression patterns between species.

### Implications for the evolution of *Schistosoma haematobium*

Our results are in line with the recent hypothesis stating that at least one introgression event occurred a long time ago between *S*. *haematobium* and *S*. *bovis* and resulted in the introgression of some *S*. *bovis* genomic tracts into several *S*. *haematobium* lineages [23]. Moreover, our study suggests that introgression resulting from past interspecific mating is observed in all of the analysed *S*. *haematobium* lineages except the allopatric lineage originating from Madagascar. Whether these isolates originate from a single or different past introgression event(s) still remains to be investigated. In the same vein, whether interspecific interactions between *S*. *haematobium* and *S*. *bovis* are still ongoing remains an open question. So far, accumulating evidence indicates that hybridisation events between *S*. *haematobium* and *S*. *bovis* are scarce even in regions where these two species live in sympatry (e.g. Senegal) [47]. We also found that all but the isolate from Madagascar in fact exhibited *S*. *bovis-*like genomic tracts within their genomes sometimes longer and in greater number than those found in the isolate from Corsica (e.g. the isolate from Mali). This result indicates that past introgression events between *S*. *haematobium* and *S*. *bovis* have not only influenced the genomic background of the parasites considered as hybrids so far but certainly concern most *S*. *haematobium* lineages across Africa. On a biological point of view, these two species use different definitive hosts, the former being associated with humans and the latter livestock. Within their respective hosts, they also differ in their tropisms (*S*. *haematobium* being urogenital and *S*. *bovis* being mesenteric). These two specific biological characteristics greatly limit possible interspecific reproduction events. Importantly however, individuals harboring both a nuclear and a mitochondrial genetic signature of *S*. *bovis* are seldom detected in humans [19,20]. More recently, individuals biologically characterized as *S*. *haematobium* (according to egg morphology and chronobiology) and genetically identified as *S*. *haematobium–bovis* hybrids were detected in cattle for the first time in Benin [48]. Together these results suggest that current simultaneous infection of animals by *S*. *haematobium* and of humans by *S*. *bovis* may occur at some level but it is not common. However, the genomic composition of those individuals from Benin has not been characterized and the possibility that these individuals in fact belong to introgressed lineages cannot be ruled out. We believe that the set of SNPs developed in this study could shed light on the genomic nature of atypical schistosome samples and provide new insights on how specific biological and genetics features are conserved within each species. The genomic pattern of the *S*. *haematobium-bovis* experimentally generated F1 hybrid observed in the current study indicates that this will be a relevant tool to detect first and early generation hybrids in natural populations. It will be of interest to combine these methods with observations on mito-nuclear profiles to clarify the extent of ongoing hybridization, changes in host specificity and potential zoonotic transmission.

Additionally, we found that *S*. *haematobium* and/or hybrid lineages also harbored different introgressed *S*. *bovis* genomic regions. In this respect, it is very likely that important diversity exists in terms of introgressed genomic regions among *S*. *haematobium* lineages in Africa and that such introgressed genes could have been maintained independently as a result of different selective pressures in different epidemiological contexts. Such diverse introgression patterns could explain some variations in virulence and compatibility with snails in *S*. *haematobium* natural populations [49,50]. We advocate that more genomic analyses are thus required to quantify such heterogeneity in introgression patterns and validate the hypothetical link between introgression patterns and these important life-history traits.

On a final note, our results also clearly indicate that the *S*. *haematobium* cluster harbors very little genetic diversity compared to *S*. *bovis* and no geographic structure was detected in *S*. *haematobium* at the African continent scale (Fig 2). These general patterns across Africa are in line with previous results obtained at a microgeographic scale based on both mitochondrial and microsatellite markers [47,51]. More generally, these results confirm, at global geographical and genomic scales, that *S*. *haematobium* is a genetically depauperate species compared to other *Schistosoma* species [51–53]. Webster et al. [52] suggested that such low genetic diversity most likely reflects a possible recent (re-)invasion of *S*. *haematobium* in Africa from a small number of founding individuals from the Arabian Peninsula and a rapid expansion from East to West. The lack of spatial structuring pattern of genetic differentiation observed in *S*. *haematobium* across Africa is in line with this hypothesis. Under such a scenario, one could emit the hypothesis that introgression events between *S*. *bovis* and *S*. *haematobium* arose before the re-colonisation of *S*. *haematobium* across Africa.

## Conclusion

Our study reveals that all the *S*. *haematobium* isolates analyzed here except the one from Madagascar, to some extent, harbor signals of introgression from *S*. *bovis* and constitute a cluster with very low genetic diversity at the African continent scale. Moreover, *S*. *haematobium* lineages differ from one another by the conserved introgressed genomic regions originating from *S*. *bovis* due either to genomic drift or local adaptive processes. This means that important variation might exist in *S*. *haematobium* populations concerning the genomic regions acquired through introgression and maintained over generations with possible different evolutionary outcomes in terms of interactions with their hosts and epidemiological dynamics. We argue that the SNPs developed in this study will constitute a toolbox for future studies aiming at better understanding the evolutionary history of the two sister species *S*. *haematobium* and *S*. *bovis* and *S*. *haematobium*-*bovis* hybrids. This approach will allow higher resolution of the genetic makeup of natural schistosome populations and will facilitate deeper insights into the epidemiology of schistosomiasis across Africa.

## Supporting information

S1 Table Sequencing and alignment statistics obtained for each isolate analysed in this study. (XLSX)

S2 Table Details of the 29 genomic tracts that display a significant decrease in genomic divergence between either at least one *S*. *haematobium* isolate and all *S*. *bovis* isolates or at least one *S*. *bovis* isolate and all *S*. *haematobium* isolates. Annotated genes that were identified in these genomic tracts are also listed.
(XLSX)

S1 Fig Plots obtained from a PCA analysis based on 56.141 SNPs developed from the initial alignment of sequencing reads on the *S*. *haematobium* genome of reference. Isolates are projected within two-dimensional spaces defined from A) axes 1 and 2; B) axes 2 and 3; C) axes 2 and 4 and D) axes 3 and 4. Isolates identified as *S*. *bovis* are in red and the F1 laboratory hybrid and isolates identified as *S*. *haematobium* are in grey.
(PDF)

S2 Fig Plots obtained from a PCA analysis based on 172.046 SNPs developed from the initial alignment of sequencing reads on the *S. bovis* genome of reference. Isolates are projected within two-dimensional spaces defined from A) axes 1 and 2; B) axes 2 and 3; C) axes 2 and 4 and D) axes 3 and 4. Isolates identified as *S*. *bovis* are in red and the F1 laboratory hybrid and isolates identified as *S*. *haematobium* are in grey.
(PDF)
