# Supplementary figures and images for: Diverging patterns of introgression from Schistosoma bovis across S. haematobium African lineages
Source: PLoS Pathog. 2021 Feb 5;17(2):e1009313. doi: 10.1371/journal.ppat.1009313 (PMC7891765; doi:10.1371/journal.ppat.1009313)

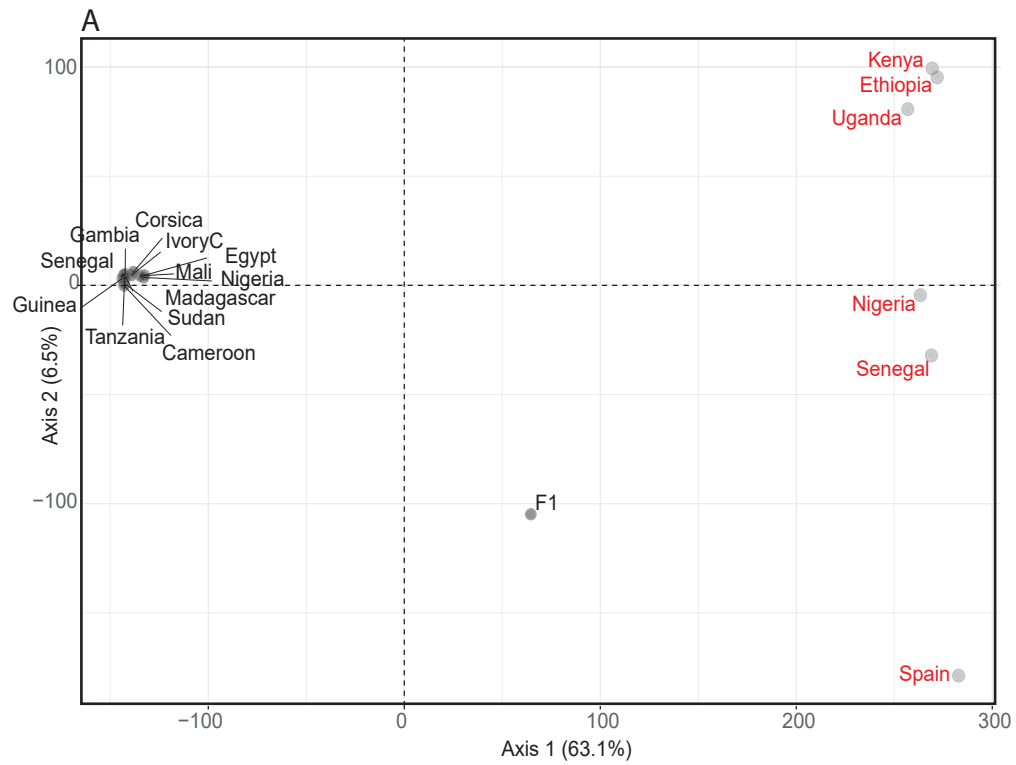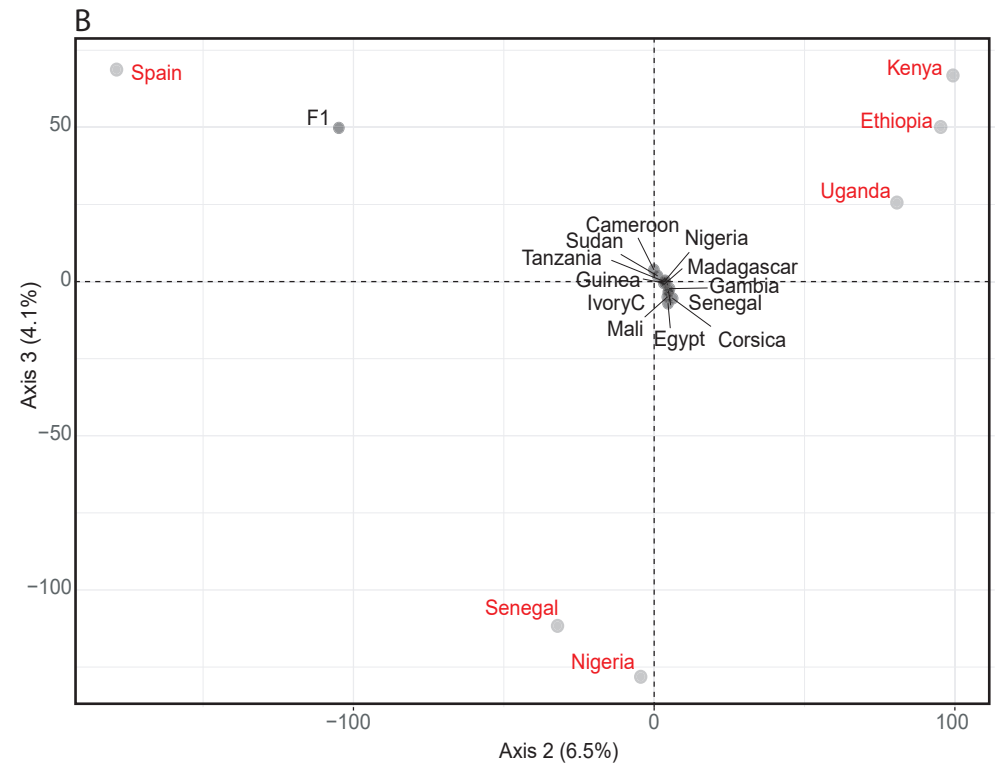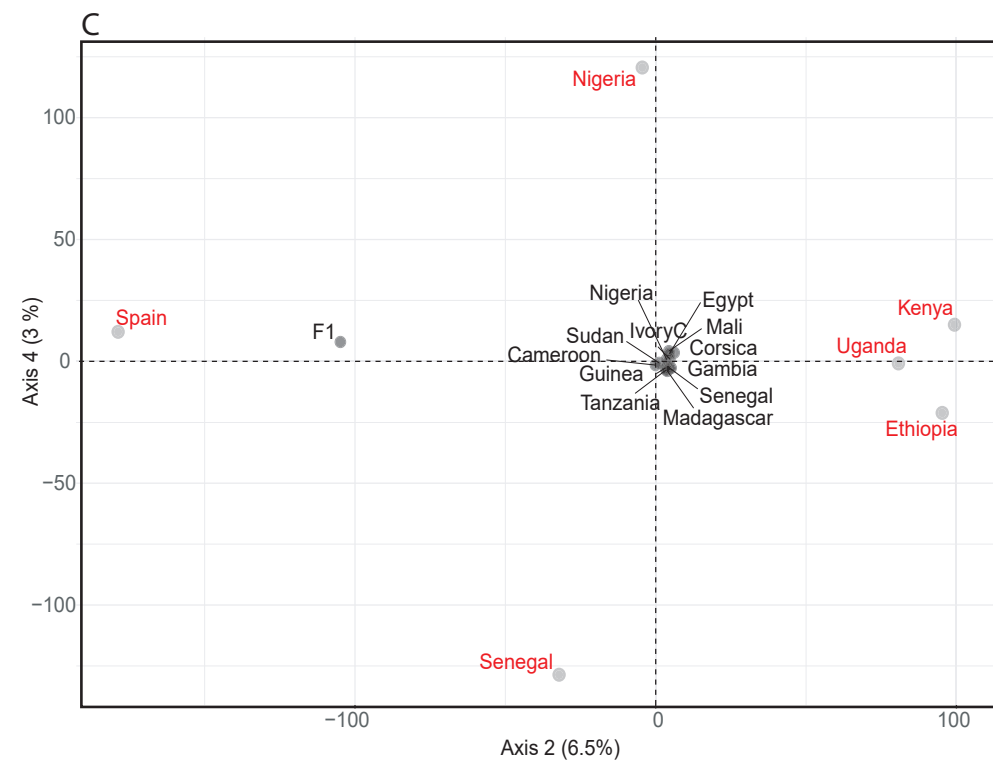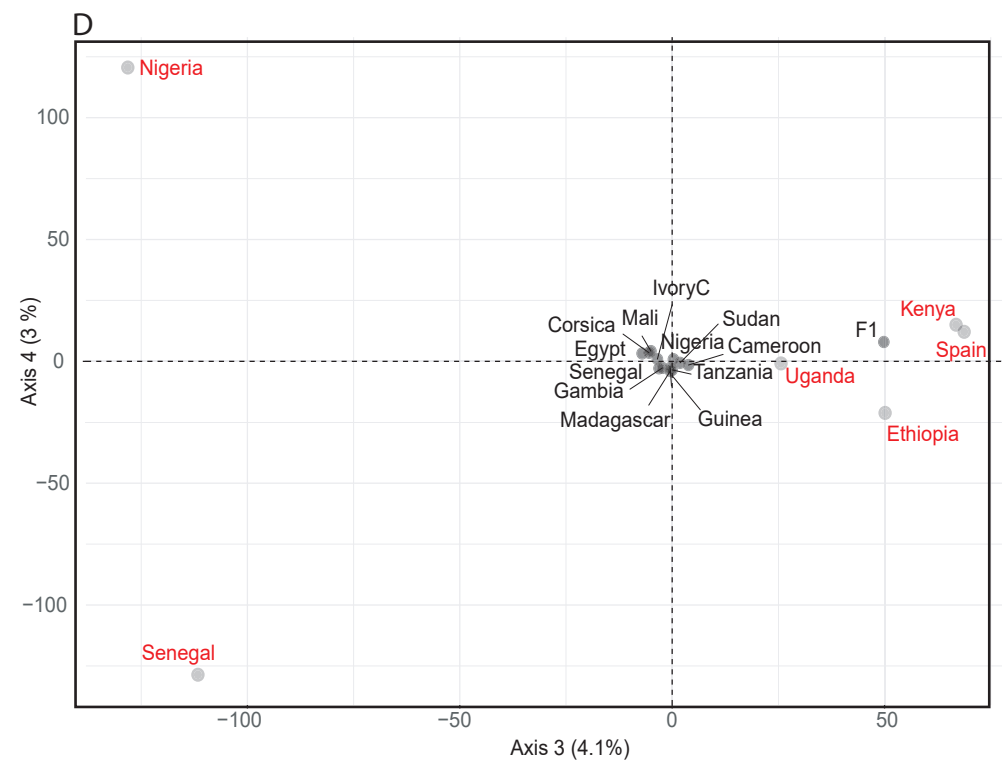

Supplement: S1 Fig — Plots obtained from a PCA analysis based on 56.141 SNPs developed from the initial alignment of sequencing reads on the S. haematobium genome of reference. Isolates are projected within two-dimensional spaces defined from A) axes 1 and 2; B) axes 2 and 3; C) axes 2 and 4 and D) axes 3 and 4. Isolates identified as S. bovis are in red and the F1 laboratory hybrid and isolates identified as S. haematobium are in grey. (PDF) [file ppat.1009313.s003.pdf]

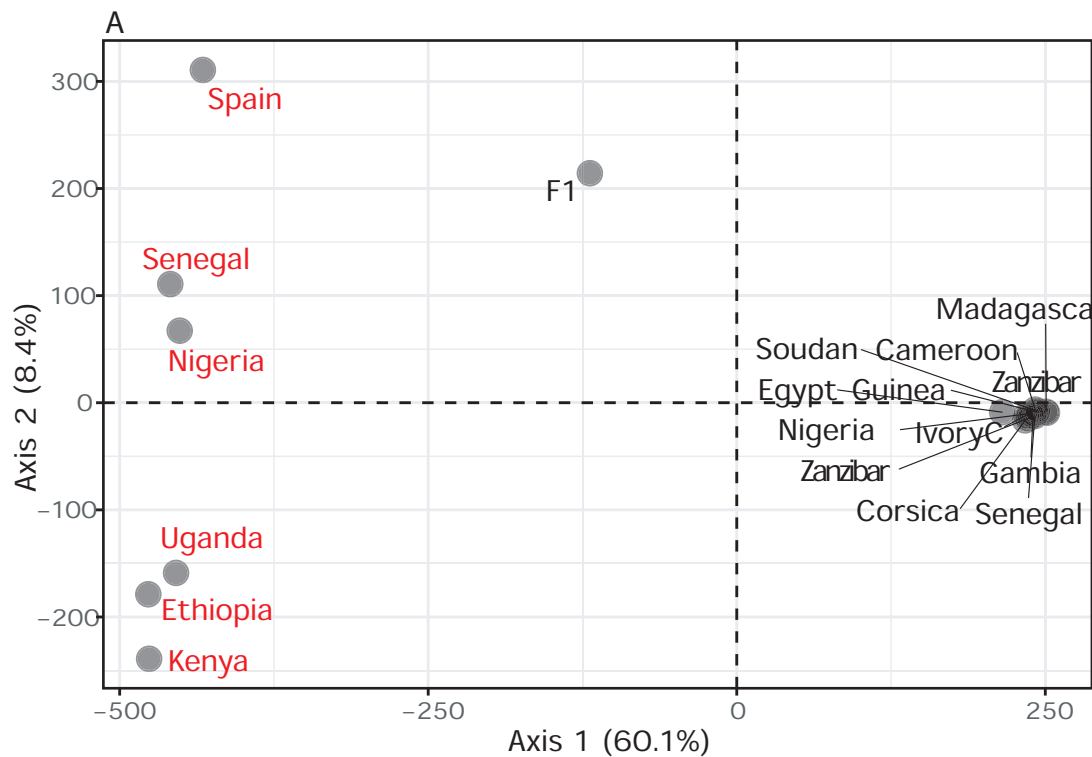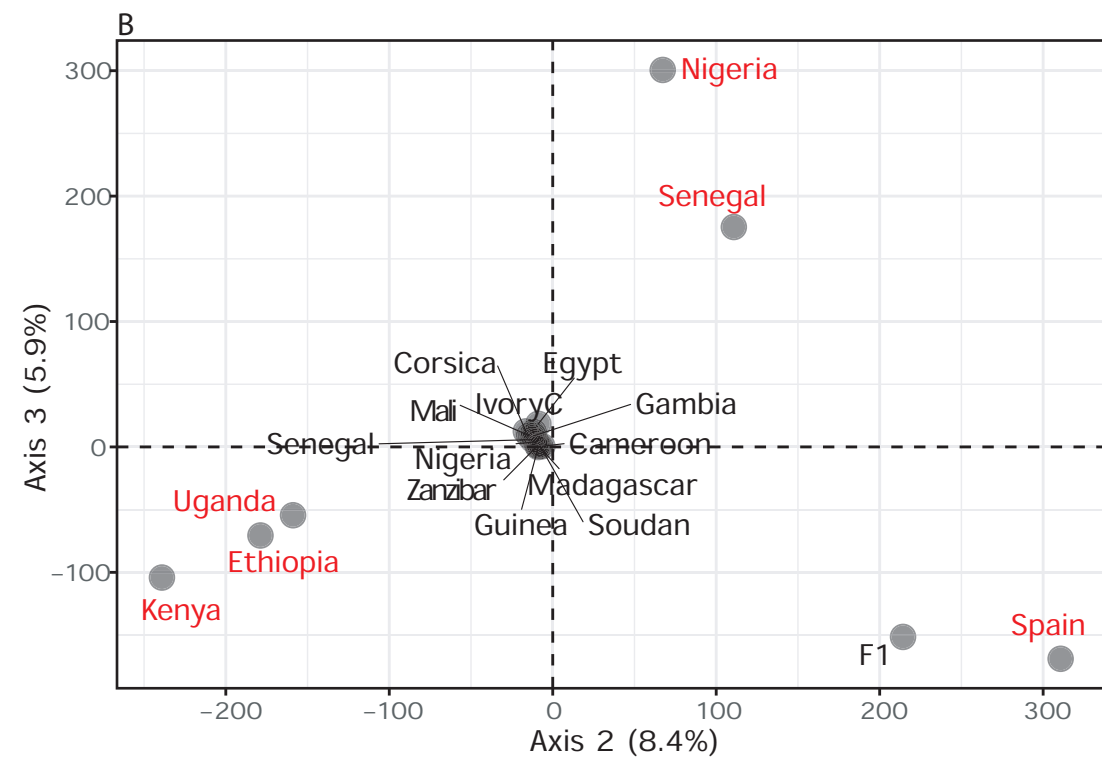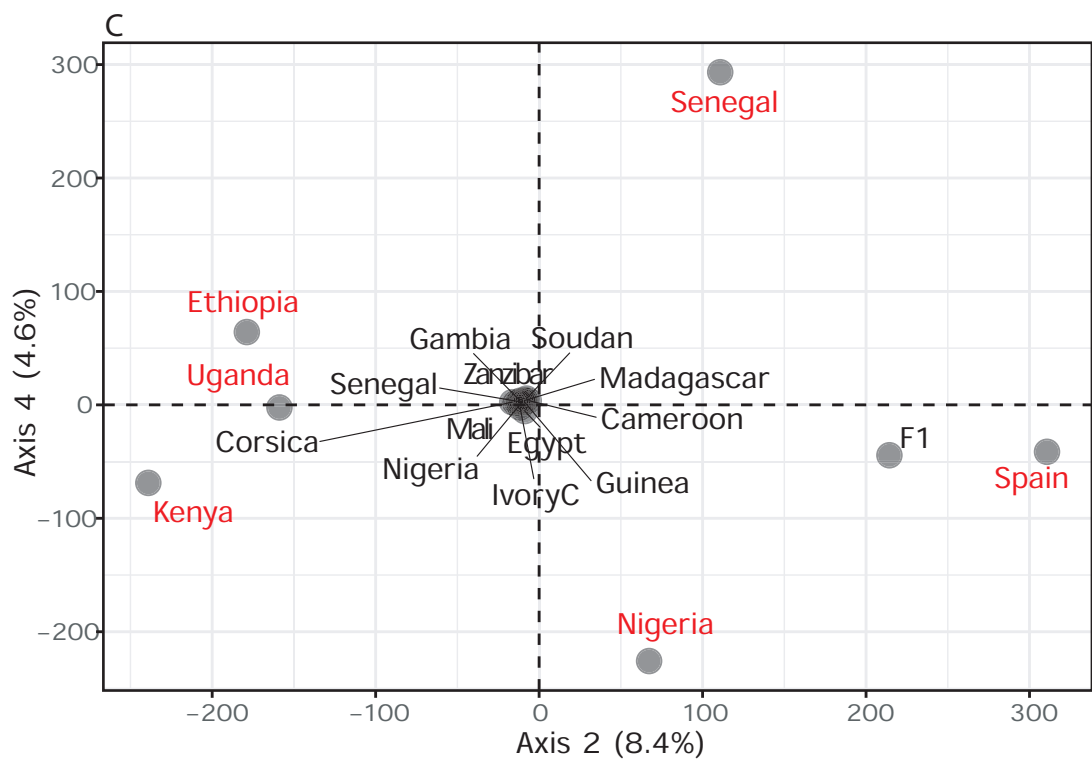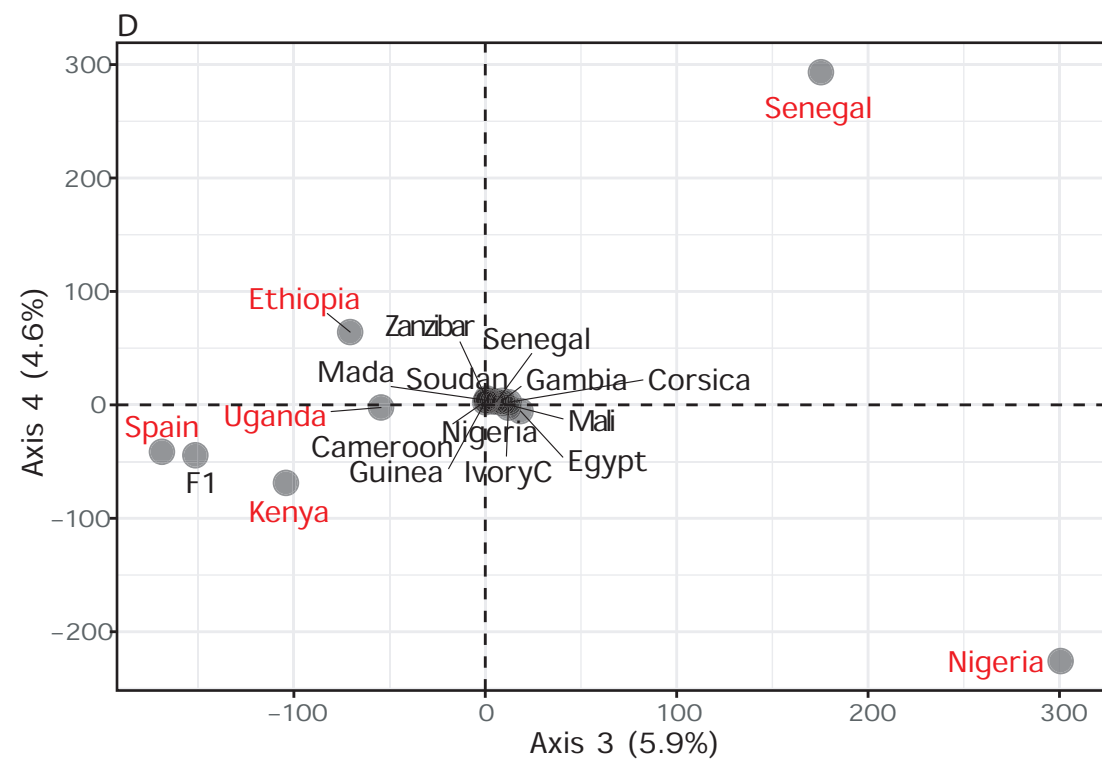

Supplement: S2 Fig — Plots obtained from a PCA analysis based on 172.046 SNPs developed from the initial alignment of sequencing reads on the S. bovis genome of reference. Isolates are projected within two-dimensional spaces defined from A) axes 1 and 2; B) axes 2 and 3; C) axes 2 and 4 and D) axes 3 and 4. Isolates identified as S. bovis are in red and the F1 laboratory hybrid and isolates identified as S. haematobium are in grey. (PDF) [file ppat.1009313.s004.pdf]
